# Efficacy and tolerability of a modified pediatric‐inspired intensive regimen for acute lymphoblastic leukemia in older adults

**DOI:** 10.1002/jha2.224

**Published:** 2021-06-22

**Authors:** Anand Ashwin Patel, Joseph Heng, Emily Dworkin, Sarah Monick, Benjamin A. Derman, Adam S. DuVall, Sandeep Gurbuxani, Satyajit Kosuri, Hongtao Liu, Michael Thirman, Lucy A. Godley, Olatoyosi Odenike, Richard A. Larson, Wendy Stock

**Affiliations:** ^1^ Department of Medicine Section of Hematology‐Oncology University of Chicago Chicago Illinois USA; ^2^ Department of Pharmacy University of Chicago Chicago Illinois USA; ^3^ Department of Medicine University of Chicago Chicago Illinois USA; ^4^ Department of Pathology Section of Hematopathology University of Chicago Chicago Illinois USA

## Abstract

Although acute lymphoblastic leukemia (ALL) is most common in pediatric and adolescent and young adult (AYA) patients, 20% of cases are diagnosed in patients ≥ 55 years old. Use of intensive pediatric regimens in AYA populations has demonstrated excellent tolerability and significant improvements in event‐free survival (EFS) and overall survival (OS). The backbone of pediatric regimens includes asparaginase and corticosteroids, both of which are associated with more toxicity in older patients and those with body mass index (BMI) ≥ 30 kg/m which leads to poor tolerance of these regimens. We tested the safety and efficacy of a dose‐modified The Cancer and Leukemia Group B 10403 regimen using reduced doses of pegylated (PEG)‐asparaginase (ASP) and corticosteroids (RD‐10403) in 30 patients with Philadelphia‐chromosome negative ALL who were ≥50‐year‐old and younger adults with significant metabolic or hepatic co‐morbidities. The complete remission rate on day 28 was 77%, 3‐year EFS was 54%, and estimated 3‐year OS was 55%. Grade 3+ toxicity was noted in 40% of patients during induction, and induction‐related mortality was 3%. Additional prospective evaluation of RD‐10403 is merited to determine efficacy and safety of this regimen and to serve as a framework for chemoimmunotherapy combination therapy.

## 1 INTRODUCTION

The estimated incidence of acute lymphoblastic leukemia (ALL) in the United States in 2021 is 5690 cases, with a median age of 15 at diagnosis; however, the incidence is bimodal, and 20% of cases occur in patients ≥ 55 years old [1, 2]. With the exception of Philadelphia‐chromosome positive ALL (Ph+ ALL), both precursor T‐cell ALL (T‐ALL) and precursor B‐cell ALL (B‐ALL) have historically been treated using similar regimens. Over the last decade, treatment paradigms for ALL in adolescents and young adults (AYAs) have shifted toward the use of intensive pediatric‐based regimens. The Cancer and Leukemia Group B (CALGB) 10403 study was a large phase II study evaluating the use of an intensive pediatric regimen in 295 adults ages 17–39 with newly diagnosed Philadelphia‐chromosome negative (Ph‐negative) ALL. Compared to historical controls, there was a significant improvement in both event‐free survival (EFS) and 3‐year overall survival (OS). Median EFS was 78.1 months, and 3‐year OS was 73% [3]. The backbone of the CALGB 10403 regimen included both pegylated‐asparaginase (PEG‐ASP) and corticosteroids. Previous analyses of AYA patients with ALL have demonstrated an improved EFS and OS associated with receipt of corticosteroids and asparaginase [4]. However, both agents also have serious toxicities that are known to increase with age and certain comorbid conditions including hyperglycemia, hepatic dysfunction, and thrombosis. When compared to patients with a body mass index (BMI) < 30 kg/m^2^, patients enrolled on CALGB 10403 with a BMI ≥ 30 kg/m^2^ also had higher rates of toxicity and significantly worse survival [5]. Obesity has previously been identified as an independent risk factor for inferior OS in adults with ALL [3, 5, 6].

Due to the treatment‐related AEs associated with intensive pediatric regimens, it has been difficult to establish a standard of care approach in adults ≥ 40 years old with newly‐diagnosed ALL [7]. Low intensity pediatric inspired regimens have demonstrated poor long‐term outcomes with survival rates < 20% in patients over the age of 60 years [8]. Unmodified pediatric inspired regimens, on the other hand, have not demonstrated improvement in the OS of older adults due to higher rates of induction‐related deaths and deaths in first complete remission (CR) [9, 10]. There are, however, emerging data suggesting that with some dosing modifications, pediatric regimens may be safely and effectively utilized in older patients with ALL [11, 12, 13].

Given our interest in exploring these issues, we tested the safety and efficacy of a modified CALGB 10403 regimen using reduced dosages of PEG‐ASP and corticosteroids (RD‐10403) in patients ≥ 50 years old and younger adults with significant metabolic or hepatic co‐morbidities. We had previously demonstrated that adequate asparaginase activity levels with less toxicity can be achieved with reduced PEG‐ASP dosing [14]. Here, we report on clinical characteristics and outcomes of patients with newly‐diagnosed Ph‐ ALL treated on RD‐10403.

## 2 METHODS

We performed a single‐center retrospective analysis of adult patients with Ph‐ ALL who were treated per RD‐10403 between August 2014 and April 2019. RD‐10403 was adopted for use at our institution starting in August 2014. April 2020 was selected as the time point for data lock to allow for at least 1 year of follow‐up. IRB approval was obtained for this study. Patients were identified by review of electronic pharmacy records, and clinical data were abstracted by manual chart review. Patients were included if they had received 250, 500, or 1000 IU/m^2^ of PEG‐ASP along with dexamethasone 10 mg/m^2^ on day 1–7 and 15–21 during induction (Tables  1A and 1B). We targeted a serum concentration of asparaginase of >0.1 IU/ml 7 days after each administration of PEG‐ASP. Dose reduction for PEG‐ASP was made for all patients ≥ 50 years old and those with BMI ≥ 30 kg/m^2^, diabetes mellitus or underlying liver dysfunction (including cirrhosis and non‐alcoholic fatty liver disease). In patients with metabolic syndromes (defined as diabetes mellitus or non‐alcoholic fatty liver disease) steroids were further reduced to administration on day 1–7 alone. Patients with CD20 positivity in ≥20% of lymphoblasts received rituximab during Course I, II, and III of therapy. Treatment continued for 2 years from the beginning of interim maintenance for women and for 3 years from the beginning of interim maintenance for men. CR was conventionally defined as the presence of ≤5% blasts in the bone marrow biopsy specimen and recovery of peripheral blood counts. Measurable residual disease (MRD) was assessed by multicolor flow cytometry on bone marrow aspirate specimens obtained at day 28 of therapy with a sensitivity of 10^−4^. High‐risk disease biology was defined as patients who had Philadelphia‐chromosome‐like (Ph‐like) ALL or an *MLL* rearrangement identified on fluorescence in‐situ hybridization or cytogenetics, *TP53* mutation detected on next‐generation sequencing assay, and/or an early T‐cell precursor ALL phenotype. Survival curves (EFS and OS) were constructed using the Kaplan‐Meier method [15]. An event was defined as the first of these: failure to achieve CR, relapse after CR, or death.

## 3 RESULTS

### 3.1 Patient characteristics and treatment doses

Thirty consecutive patients with Ph‐ B‐ALL or T‐ALL that were treated with RD‐10403 from August 2014 to April 2019 were identified. Patient characteristics are outlined in Table 2. The median age of this cohort was 46 years old (25%–75% interquartile range (IQR), 33–60); sixteen patients were ≥ 40 years old with the oldest patient being 76 years old. Median BMI was 28 (25%–75% IQR, 24–33), 23% of patients had diabetes mellitus, and 17% had underlying liver disease (two patients had non‐alcoholic fatty liver disease, two had chronic liver disease secondary to previous hepatitis C infection, and one had drug‐induced liver injury). Seventy percent of patients had pre‐B‐ALL and 30% had T‐ALL; 53% of patients were identified as having high‐risk disease biology. The median follow‐up time was 1.69 years (range 0.06–5.64 years). The median PEG‐ASP dose during induction was 1000 IU/m^2^, and 83% of patients achieved therapeutic asparaginase levels at day 11 of induction (≥0.1 IU/ml). Thirteen patients received 1000 IU/m^2^, 12 patients received 500 IU/m^2^, and four patients received 250 IU/m^2^. One patient had PEG‐ASP held during induction due to drug‐induced hepatotoxicity from home medications. Of the four additional patients that did not achieve therapeutic PEG‐ASP levels at day 11, three received 500 IU/m^2^, and one received 250 IU/m^2^. No patients were given a second dose of PEG‐ASP during induction. The median total dexamethasone dose during induction was 140 mg/m^2^; 22 patients received 10 mg/m^2^ on day 1–7 and 15–21 of induction, seven received 10 mg/m^2^ on day 1–7 of induction, and one received 10 mg/m^2^ on day 1–7 and 15–18 of induction.

**TABLE 2:** Patient characteristics

Patient characteristics (*N* = 30)	
Age, median (25%–75% IQR)	46 (33–60)
Male gender (%)	47%
BMI, median (25‐75% IQR)	28 (24–33)
Underlying liver disease	17%
Diabetes mellitus	23%

Phenotype (*n* = 30)	
B‐cell, *n* (%)	21 (70%)
T‐cell, *n* (%)	9 (30%)

High‐risk disease biology (*n* = 16)	
Ph‐like	4 (25%)
*MLL*‐rearranged	3 (19%)
*TP53*‐mutated	5 (31%)
ETP ALL	3 (19%)

Treatment dosing	
Dexamethasone induction dose (mg/m^2^), median (25‐75% IQR)	140 (113–140)
PEG‐ASP induction dose (IU/m^2^), median (25‐75% IQR)	500 (500–1000)

Abbreviations: BMI, body mass index; ETP, early T‐cell precursor; IQR, interquartile range; PEG‐ASP, peg‐asparaginase; Ph‐like, Philadelphia‐chromosome like.

### 3.2 Treatment outcomes

**FIGURE 1:**
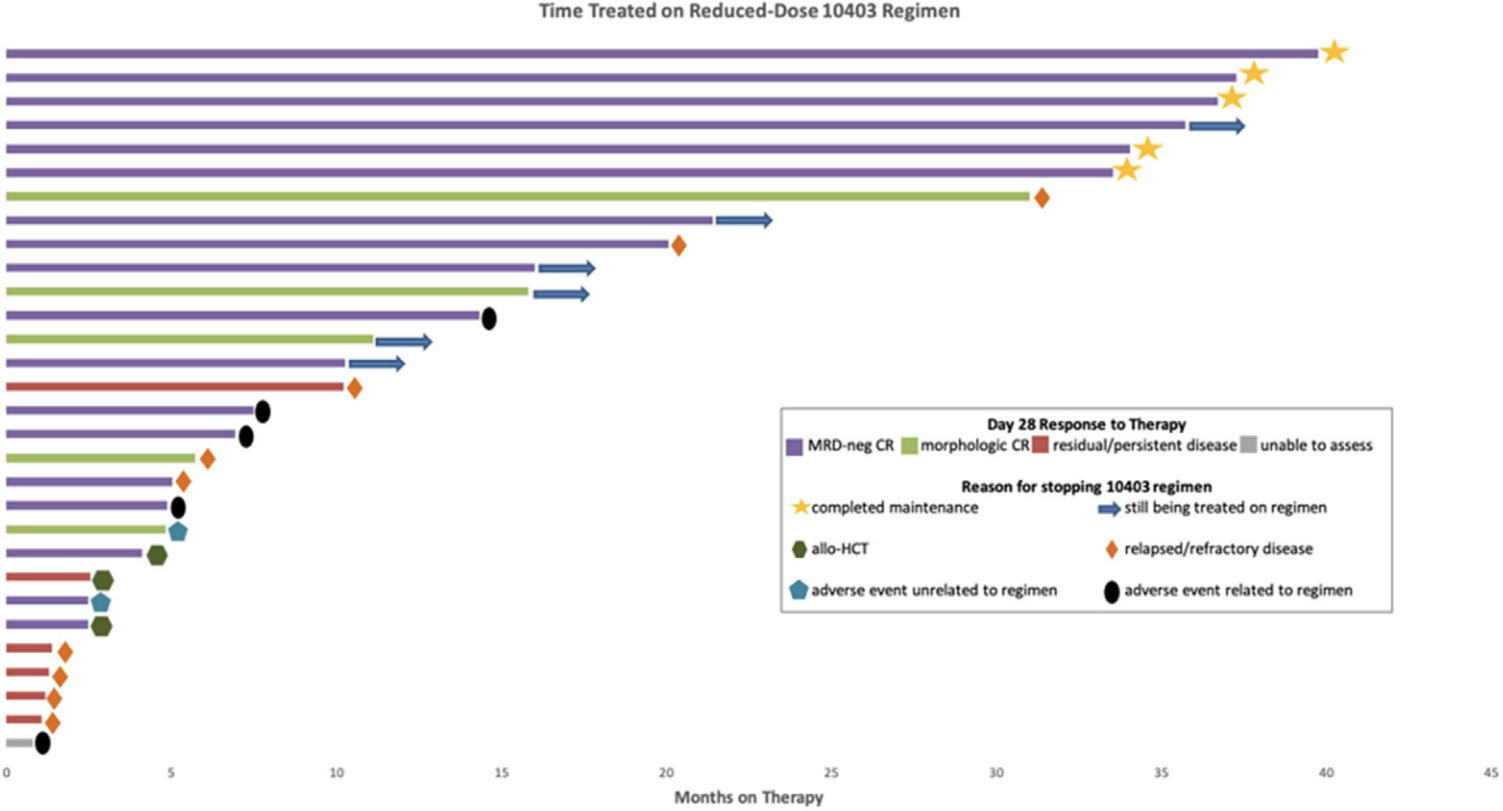
Duration of treatment on Reduced‐Dose 10403 Regimen Abbreviations: CR, complete remission; allo‐HCT, allogeneic hematopoietic stem cell transplantation; MRD, measurable residual disease.

**FIGURE 2:**
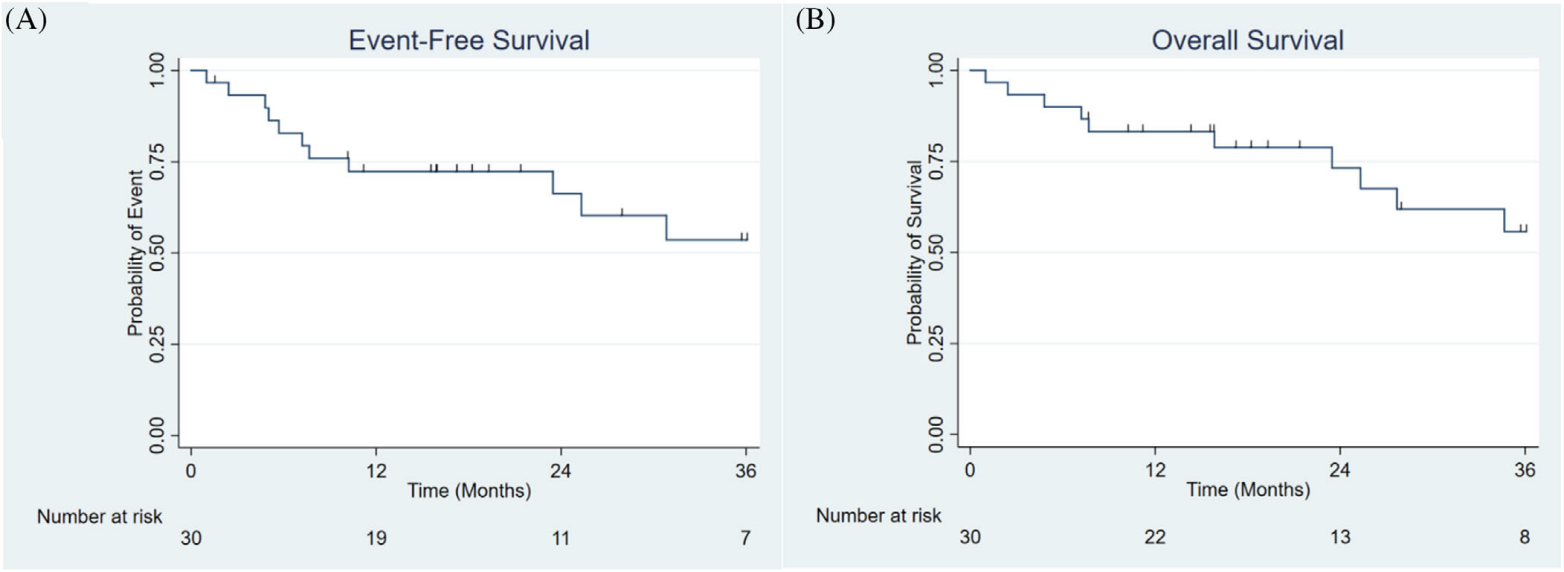
Survival outcomes of patients receiving RD‐10403. (A) Three‐year event‐free survival of patients receiving reduced‐dose 10403. (B) Three‐year overall survival of patients receiving reduced‐dose 10403

**FIGURE 3:**
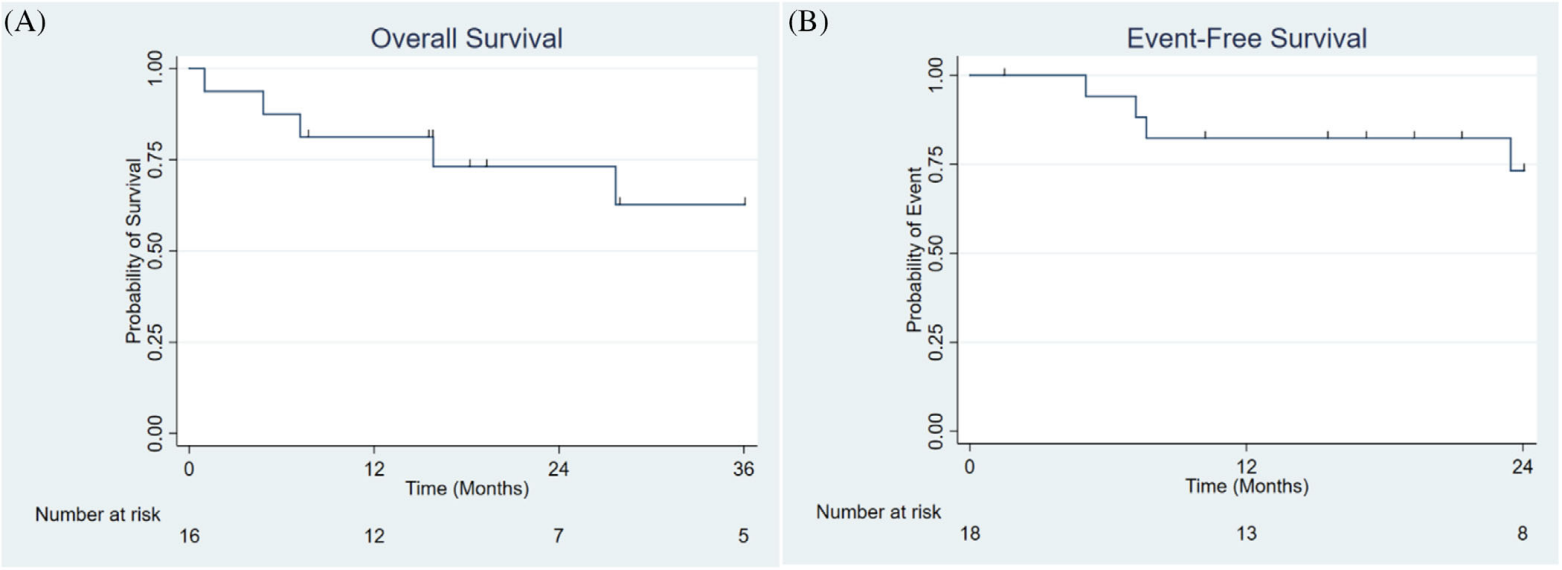
Outcomes of patients with high‐risk disease biology and patients with MRD negativity after induction. (A) Three‐year overall survival of patients with high‐risk disease biology. (B) Two‐year event‐free survival of patients that achieved undetectable measurable residual disease by day 28

Treatment and response characteristics for each patient are summarized in Figure 1. The CR rate at 28 days after starting induction therapy (Day 28) was 77% (95% CI, 61%–93%). Of the 23 patients with CR, 18 patients achieved MRD negativity as detected by 10‐color flow cytometry (77%, 95% CI 60%–94%). Of the 16 patients with high‐risk disease biology, the day 28 CR rate was 69% (*n* = 11), and nine patients achieved MRD negativity. At time of data lock, six patients were still being treated on the regimen, five had completed maintenance therapy per RD‐10403, five had discontinued due to adverse events (AEs) attributable to RD‐10403, and two had discontinued due to AEs unrelated to RD 10403 (one patient developed ischemic cardiomyopathy, and one patient had exacerbation of a previously diagnosed bipolar disorder). Three patients underwent allogeneic hematopoietic stem cell transplantation (allo‐HCT) in first CR (two patients with *MLL* rearrangement, one with persistent MRD positivity). Nine patients (30%) had discontinued RD‐10403 due to relapsed/refractory disease. For these nine, a median of one salvage therapy was utilized (range 1–4), and five patients subsequently underwent allo‐HCT in a second CR or later. For the entire cohort, the estimated 3‐year EFS was 54% (95% CI, 30%–73%), and estimated 3‐year OS was 55% (95% CI, 31%–74%) (Figures 2A and 2B). The estimated 3‐year OS for the 16 patients with high‐risk disease biology was 61% (95% CI, 29%–83%) (Figure 3A). Due to shorter follow‐up for patients with MRD negativity at day 28, 2‐year EFS was analyzed and was estimated to be 76% (95% CI, 48%–90%) (Figure 3B). Thirteen patients had a BMI ≥ 30 kg/m^2^ at the beginning of induction therapy; day 28 CR rate was 69%, and six patients achieved MRD negativity at day 28. Three patients completed maintenance therapy, three patients were still being treated on RD‐10403 at time of data lock, four patients had relapsed/refractory disease, one patient underwent allo‐HCT, one patient discontinued RD‐10403 due to toxicity attributable to the regimen, and one patient discontinued RD‐10403 due to toxicity unrelated to the regimen. Estimated 3‐year OS was 46% for these 13 patients.

### 3.3 Treatment‐related AEs

At least one Grade 3+ non‐hematologic AE was seen in 40% of patients during induction (hepatotoxicity in eight patients, thrombosis in four, pancreatitis in two). Regarding the four patients with thrombosis, all four patients developed upper extremity deep vein thrombosis (DVT). One patient had proximal DVTs in both lower extremities as well. All patients received therapeutic anticoagulation for a minimum of 6 months with interruptions of anticoagulation when platelet count was ≤50000/μl. There was one Grade 5 death due to septic shock (Table 3). Among the 12 patients with Grade 3+ AE during induction, nine received subsequent PEG‐ASP at the same dose during consolidation, with four of them developing a Grade 3+ toxicity during consolidation. Of these four patients, one patient received PEG‐ASP through the end of interim maintenance but did not continue further due to recurrent hyperbilirubinemia, one patient received PEG‐ASP through the end of delayed intensification, one received PEG‐ASP with all courses of RD‐10403 leading up to allo‐HCT, and one patient did not receive additional PEG‐ASP due to anaphylaxis during consolidation and was switched to *Erwinia* asparaginase for subsequent doses. When comparing patients who developed Grade 3+ AE during induction to the patients who did not, neither BMI (medians, 26.8 vs. 27.8, *p* = 0.95) nor the age at time of induction (medians, 42 years old vs. 49 years old, *p* = 0.85) were significantly different.

**TABLE 3:** Toxicities of RD‐10403

Grade 3+ toxicities during induction (*n* = 30)	
Hepatotoxicity, *n* (%)	8 (27%)
Thrombosis, *n* (%)	4 (13%)
Pancreatitis, *n* (%)	2 (7%)
Septic Shock, *n* (%)	1 (3%)

Causes of treatment‐related mortality beyond induction (*n* = 2)	
Septic shock, *n*	1
Hepatic failure, *n*	1

**TABLE 1A:** Comparison of peg‐asparaginase and steroid dosing in the standard CALGB 10403 regimen and the reduced‐dose regimen

CALGB 10403 dosing for PEG‐ASP and corticosteroids		
Agent	Standard dose	Reduced dose
PEG‐ASP (Throughout treatment course)	2500 IU/m^2^ IV	1000 IU/m^2^ IV ^*^Can further reduce for Age ≥ 50 years or comorbidities of NASH, diabetes, or obesity
Corticosteroids (During Induction)	Prednisone 60 mg/m2/day PO or IV on days 1–28 ^*^Equivalent to dexamethasone 252 mg/m^2^/induction course	Dexamethasone 10 mg/m^2^ PO or IV on days 1–7 and 15–21 ^*^Can further reduce to 10 mg/m^2^ on Days 1–7 alone

Abbreviations: IU, international units; IV, intravenously; PEG‐ASP, peg‐asparaginase; PO, orally.

**TABLE 1B:** Treatment schema for RD‐10403

RD‐10403 regimen	
Course I Remission induction	·Vincristine 1.5 mg/m^2^ (max 2 mg) IV on days 1, 8, 15 and 22 ·Daunorubicin 25 mg/m^2^ IV on days 1, 8, 15 and 22 ·Dexamethasone 10 mg/m^2^ PO/IV daily on days 1–7 and 15–21 ·PEG‐ASP 1000 units/m^2^ IV on day 4 ·Cytarabine 70 mg IT on day 1 ·Methotrexate 15 mg IT on day 8 and 29 (for CNS+ disease also give on day 15 and 22)
Course II Remission consolidation	·Cyclophosphamide 1000 mg/m^2^ IV on day 1 and 29 ·Cytarabine 75 mg/m^2^ SUBQ/IV on days 1–4, 8–11, 29–32 and 36–39 ·Mercaptopurine 60 mg/m^2^ PO on days 1–14 and 29–42 ·Vincristine 1.5 mg/m^2^ (max 2 mg) IV on days 15, 22, 43 and 50 PEG‐ASP 1000 units/m^2^ IV on day 15 and 43 ·Methotrexate 15 mg IT on days 1, 8, 15 and 22 (for CNS3 omit on day 15 and 22)
Course III Interim maintenance	·Vincristine 1.5 mg/m^2^ (max 2 mg) IV on days 1, 11, 21,31 and 41 ·PEG‐ASP 1000 units/m^2^ IV on days 2 and 22 ·Methotrexate 100 mg/m^2^ IV on day 1 ·Methotrexate 150 mg/m^2^ IV on day 11 ·Methotrexate 200 mg/m^2^ IV on day 21 ·Methotrexate 250 mg/m^2^ IV on day 31 ·Methotrexate 300 mg/m^2^ IV on day 41 ·Methotrexate 15 mg IT on day 1 and 31
Course IV Delayed intensification	·Vincristine 1.5 mg/m^2^ (max 2 mg) IV on day 1, 8, 15, 43 and 50 ·Doxorubicin 25 mg/m^2^ IV on day 1, 8 and 15 ·Dexamethasone 5 mg/m^2^ PO twice daily on day 1–7 and 15–21 ·PEG‐ASP 1000 units/m^2^ IV on day 4 and 43 ·Cyclophosphamide 1000 mg/m^2^ IV on day 29 ·Cytarabine 75 mg/m^2^ SUBQ/IV on days 29–32 and 36–39 ·Thioguanine 60 mg/m^2^ PO on day 29–42 ·Methotrexate 15 mg IT on day 1, 29 and 36
Course V Maintenance therapy	·Vincristine 1.5 mg/m^2^ (max 2 mg) on day 1, 29 and 57 ·Dexamethasone 3 mg/m^2^ PO twice daily on days 1–5, 29–33 and 57–61 ·Mercaptopurine* 75 mg/m^2^ PO daily on days 1–84 ·Methotrexate* 20 mg/m^2^ PO on day 8, 15, 22, 29, 36, 43, 50, 57, 64, 71 and 78 ·Methotrexate 15 mg IT on day 1 and 29 (omit day 29 for cycle 5 and beyond) *Repeated every 84 days for a total of 2 years for females and 3 years for males from the start of interim maintenance*

For patients with CD20 positivity on ≥20% of blasts rituximab 375 mg/m^2^ is given on day 2 and 16 during course I, day 1, 8, 29, and 36 of course II and day 1 and 11 of course III.

Abbreviations: CNS, central nervous system; IT, intrathecal; IV, intravenous; PEG‐ASP, peg‐asparaginase; PO, oral; SUBQ, subcutaneous.

* Mercaptopurine and oral methotrexate doses are titrated to maintain a platelet count > 75,000/μl and absolute neutrophil count between 750–1500/μl.

The median asparaginase level for the entire cohort was 0.14 IU/ml on day 11 (7 days after the initial dose of PEG‐ASP). The median day 11 asparaginase level was significantly higher in patients who developed Grade 3+ AE (0.22 IU/ml, IQR 0.15–0.26) when compared to those without Grade 3+ AE (0.12 IU/ml, IQR 0.1–0.17) (*p* = 0.013). Two additional treatment‐related deaths occurred beyond induction therapy (one due to septic shock and one due to asparaginase‐induced hepatic failure), for an overall treatment‐related mortality (TRM) of 10%. Of the two additional patients that discontinued RD‐10403 due to attributable AEs, one had developed persistent Grade 1 neuropathy, and the other was diagnosed with methotrexate‐associated cirrhotic liver disease.

## 4 DISCUSSION

Given that fewer than 20% of older adults with ALL are long‐term survivors, improving upon the standard of care in this population is of essential importance [2]. A number of asparaginase free regimens have been studied in this patient population and have demonstrated high CR rates but poor long‐term outcomes [16, 17, 18, 19]. Overall these regimens were found to have toxicities in keeping with their respective components, with the achievement of some durable responses but with considerable room for improvement.

Recent data have demonstrated that asparaginase cannot only be safely incorporated into ALL treatment regimens for older adults, but that it may also lead to durable long‐term outcomes [11, 12, 13]. In this study we show that the RD‐10403 regimen is feasible both in older adults up to the age of 76 and in patients with metabolic and/or hepatic comorbidities. The day 28 CR rate was 77% with this regimen with an MRD‐negativity rate of 77% in those that achieved a CR. Furthermore, estimated 3‐year EFS was 54%, and estimated 3‐year OS was 55% for the entire cohort, with only four patients receiving an allo‐HCT in CR1. This regimen was effective in patients with high‐risk disease biology as well with a day 28 CR rate of 69% and an estimated 3‐year OS of 58%. This stands in contrast to findings from the GRAAL‐2005 study, which found significantly worse survival and poor tolerability of a pediatric‐inspired protocol in patients aged 55–60 with newly‐diagnosed ALL [10]. Estimated 3‐year OS for patients with BMI ≥ 30 kg/m^2^ was 46% while the estimated 3‐year OS for patients with BMI < 30 kg/m^2^ was 61%; however, the difference in OS was not statistically significant. This is consistent with previous analyses, including the AYA patients enrolled on CALGB 10403, identifying obesity as a risk factor for poorer OS [3, 5, 6].

Despite the encouraging long‐term outcomes seen in our high‐risk cohort compared to historical data for similar patients, it is important to compare the toxicities of the RD‐10403 with the toxicity data reported from the CALGB 10403 study upon which it was modeled. Specific grade 3 of 4 AEs observed in the patients enrolled on CALGB 10403 included hyperglycemia (31%), alanine transaminase elevation (29%), febrile neutropenia (24%), hyperbilirubinemia (19%), aspartate transaminase elevation (13%), and thrombosis (5%) with a TRM of 2% during induction. In comparison, 40% percent of our RD‐10403 patients had at least one Grade 3+ AE during induction with TRM of 3%. Nine of the 12 patients with Grade 3 of 4 AEs were able to receive additional PEG‐ASP during post‐remission therapy. Overall TRM attributable to RD‐10403 was 10%, and two (6%) patients were not continued on the regimen due to toxicity. We did not find a significant difference in BMI or age between patients who had Grade 3+ AEs and those who did not. However, median day 11 asparaginase level was significantly higher in those with Grade 3+ AEs. Previous work by Derman et al and by others has demonstrated lower rates of Grade 3+ AEs in patients receiving a reduced dose of asparaginase in comparison to a standard dose, suggesting that dose reduction may be critical in allowing for safe administration of this regimen to older patients [14, 20, 21]. Adequate asparagine depletion has been demonstrated to improve outcomes in ALL and 83% of patients in our cohort achieved the recommended level of asparaginase activity during induction [22]. Furthermore, prospective data from the NOPHO ALL2008 trial in pediatric patients with ALL have demonstrated that lower doses of asparaginase can significantly reduce toxicity while preserving efficacy and survival outcomes [21]. Limitations of our study include its retrospective nature, the small sample size and the fact that this was a single‐center experience. In addition, 5‐year follow‐up has not yet been achieved for the majority of this cohort.

It is also important to consider that the entire traditional therapeutic approach for treatment of ALL, particularly for older adults, is undergoing re‐evaluation in new frontline trials and perhaps heralds a new era that introduces new agents in the frontline and minimizes exposure to the traditional chemotherapeutics that have been the backbone of the pediatric regimens. The FDA approvals of inotuzumab ozogamicin (InO) in relapsed or refractory (R/R) ALL and blinatumomab in R/R ALL and in MRD+ ALL have given clinicians targeted therapies with remarkable efficacy and tolerability [23, 24, 25]. These targeted therapies are being evaluated in the frontline setting as well. Phase II data on blinatumomab induction followed by POMP maintenance in newly‐diagnosed patients with Ph‐ B‐ALL ≥ 65 years old was notable for an overall response rate (ORR) of 66%, no treatment‐related deaths, and 1‐year OS of 65%. The most common grade 3+ non‐hematologic toxicities were hyperglycemia (14%), dyspnea (10%), febrile neutropenia (10%), and hypertension (10%) [26]. A single‐center Phase II trial in newly‐diagnosed Ph‐ B‐ALL patients ≥ 60 years old investigating InO in combination with reduced‐intensity chemotherapy demonstrated an ORR of 98%. The estimated 3‐year OS was 56%, similar to what was noted in our cohort of patients treated with RD‐10403. It is important to note that there were significant toxicities noted with this regimen, including Grade 3+ hepatic AEs in 33% of patients and 12% TRM.[27] Stelljes and colleagues reported on a Phase II study evaluating InO induction therapy followed by a conventional chemotherapy‐based consolidation including asparaginase and subsequent maintenance therapy. An interim analysis was significant for a CR/ CR with incomplete hematologic recovery (CRi) rate of 100% with a 1‐year OS of 87%. Non‐hematologic grade 3+ toxicities during cycle 1 of induction included hyperglycemia (19%), hyperbilirubinemia (10%), and infection (8%). Of the 31 patients assessed for response there have also been two deaths in patients in first remission [28]. Longer follow‐up of these novel reduced intensity or “chemotherapy‐free” approaches are needed to ensure their efficacy and safety. In addition, the same resistance mechanisms reported with use of blinatumomab and InO in the relapsed and refractory setting (loss of CD19 or CD22 expression, lineage switch at relapse of disease, T‐cell exhaustion) may be seen in the frontline setting and necessitate combination therapy approaches [29, 30, 31, 32]. RD 10403 may be a backbone frontline therapy for non‐AYA adults with ALL that either blinatumomab or InO can be layered onto similar to what is being tested in ongoing randomized frontline trials (NCT02003222, NCT03150693).

The feasibility of regimens similar to RD‐10403 may be of particular relevance for the many regions of the world where access to new antibody therapies remains very limited and cost‐prohibitive. In addition, the current treatment paradigm for T‐ALL still lacks targeted therapies, although some new agents are being actively investigated in the R/R setting [33]. Therefore, given the efficacy that we report here in this older and high‐risk population, the critical role of asparaginase and corticosteroids in improving survival of children and young adults with ALL, the ability to achieve therapeutic levels with lower doses of asparaginase and the poor outcomes of obese patients in the AYA population when full dose pediatric regimens are employed, we believe that there is still a strong rationale for prospective trials testing‐modified pediatric intensive regimens with dose reduced asparaginase in these high‐risk populations with ALL.

## AUTHOR CONTRIBUTIONS

Anand Ashwin Patel designed the research study, analyzed data, and wrote the paper. Joseph Heng, Emily Dworkin, and Sarah Monick analyzed the data, helped to draft the paper, and provided critical revisions. Adam S. DuVall, Sandeep Gurbuxani, Satyajit Kosuri, Benjamin A. Derman, Hongtao Liu, Michael Thirman, Lucy A. Godley, Olatoyosi Odenike, and Richard A. Larson helped to draft the paper and provided critical revisions. Wendy Stock designed the research study, wrote the paper, and provided critical revisions.

## CONFLICT OF INTEREST

Anand Ashwin Patel, Sarah Monick, Adam S. DuVall, Sandeep Gurbuxani, and Satyajit Kosuri have no conflict of interest. Joseph Heng is honoraria from OncLive, while Emily Dworkin is honoraria from Abbvie. Benjamin A. Derman is in advisory board from Sanofi. Hongtao Liu did research funding from BMS and Karyopharm; is honoraria from Agios. Michael Thirman did research funding from Gilead Sciences, Pharmacyclics, Janssen, AbbVie, Merck, Syndax, TG Therapeutics, Tolero; is in advisory board from AstraZeneca, Celgene, Roche/Genentech, Pharmacyclics, Janssen, Abbvie. Lucy A. Godley is advisory board from Invitae, Inc; royalties from UpToDate. Olatoyosi Odenike did research funding from AstraZeneca, ABBVIE, astex, agios, incyte, janssen, NS‐Pharma, Oncotherapy Sciences, BMS; is honoraria/has ad board membership‐ ABBVIE, BMS, Celgene, Novartis, Taiho, PRA. Richard A. Larson has acted as a consultant or advisor to Amgen, Ariad/Takeda, Celgene/Bristol Myers Squibb, CVS/Caremark, Epizyme, MorphoSys, and Novartis, and has received clinical research support to his institution from Astellas, Celgene, Cellectis, Daiichi Sankyo, Forty Seven/Gilead, Novartis, Rafael Pharmaceuticals, and royalties from UpToDate. Wendy Stock is Honoraria from Abbvie, Jazz, Kite, Morphosys, Pfizer, Servier.

## Data Availability

Data are available on request from the corresponding author.
